# Supplementary material for: Comparative Evaluation of Vitek 2 and Etest versus Broth Microdilution for Ceftazidime/Avibactam and Ceftolozane/Tazobactam Susceptibility Testing of Enterobacterales and Pseudomonas aeruginosa
Source: Antibiotics (Basel). 2022 Jun 27;11(7):865. doi: 10.3390/antibiotics11070865 (PMC9312067; doi:10.3390/antibiotics11070865)
Supplement: Supplementary file 1 [file antibiotics-11-00865-s001.zip › antibiotics-1752611-supplementary.pdf]

Table S1. Comparison of testing methods for the determination of susceptibility of challenge as well as ESBL-negative and carbapenem-susceptible isolates to ceftazidime/avibactam according to the current EUCAST breakpoints.

Organism (No of isolates)	Assay	Resistance rate	MIC (mg/L)		
			Range	MIC_{50}	MIC_{90}
***Enterobacterales* (*n* = 83)**	BMD	23%	≤0.125 - >16	1	>16
K. pneumoniae (*n* = 57)		30%	≤0.125 - >16	1	>16
Challenge (*n* = 44)		36%	≤0.125 - >16	1	>16
ESBL-negative, carbapenem-susceptible (*n* = 13)		0%	≤0.125 - 0.5	≤0.125	0.5
E. coli (*n* = 19)		5%	≤0.125 - >16	≤0.125	0.25
Challenge (*n* = 11)		9%	≤0.125 - >16	≤0.125	0.25
ESBL-negative, carbapenem-susceptible (n = 8)		0%	≤0.125 - ≤0.125	≤0.125	≤0.125
E. cloacae (*n* = 7)		29%	≤0.125 - >16	≤0.125	>16
Challenge (*n* = 3)		67%	0.25 - >16	>16	>16
ESBL-negative, carbapenem-susceptible (*n* = 4)		0%	≤0.125 - ≤0.125	≤0.125	≤0.125
***Enterobacterales* (*n* = 83)**	Vitek 2	22%	≤0.125 - >8	0.5	>8
K. pneumoniae (*n* = 57)		28%	≤0.125 - >8	1	>8
Challenge (*n* = 44)		34%	≤0.125 - >8	1	>8
ESBL-negative, carbapenem-susceptible (*n* = 13)		0%	≤0.125 - 0.5	≤0.125	0.5
E. coli (*n* = 19)		5%	≤0.125 - >8	≤0.125	0.25
Challenge (*n* = 11)		9%	≤0.125 - >8	≤0.125	≤0.125
ESBL-negative, carbapenem-susceptible (n = 8)		0%	≤0.125 - 0.25	≤0.125	0.25
E. cloacae (*n* = 7)		29%	≤0.125 - >8	0.25	>8
Challenge (*n* = 3)		67%	0.5 - >8	>8	>8
ESBL-negative, carbapenem-susceptible (*n* = 4)		0%	≤0.125 - 0.25	0.25	0.25
***Enterobacterales* (*n* = 83)**	Etest	23%	0.03 - >256	1	>256
K. pneumoniae (*n* = 57)		30%	0.125 - >256	1	>256
Challenge (*n* = 44)		36%	0.125 - >256	2	>256
ESBL-negative, carbapenem-susceptible (*n* = 13)		0%	0.125 - 1	0.125	1
E. coli (*n* = 19)		5%	0.03 - >256	0.125	0.25
Challenge (*n* = 11)		9%	0.06 - >256	0.25	0.5
ESBL-negative, carbapenem-susceptible (n = 8)		0%	0.03 - 0.125	0.06	0.125
E. cloacae (*n* = 7)		29%	0.25 - >256	0.25	>256
Challenge (*n* = 3)		67%	1 - >256	>256	>256
ESBL-negative, carbapenem-susceptible (*n* = 4)		0%	0.25 - 0.25	0.25	0.25
***P. aeruginosa* (*n* = 17)**	BMD	29%	1 - >16	2	>16
Challenge (*n* = 11)		45%	2 - >16	8	>16
ESBL-negative, carbapenem-susceptible (*n* = 6)		0%	1 - 4	2	4
***P. aeruginosa* (*n* = 17)**	Vitek 2	29%	1 - >8	2	>8
Challenge (*n* = 11)		45%	2 - >8	8	>8
ESBL-negative, carbapenem-susceptible (*n* = 6)		0%	1 - 2	2	2
***P. aeruginosa* (*n* = 17)**	Etest	24%	1 - 256	2	128
Challenge (*n* = 11)		36%	2 - 256	4	128
ESBL-negative, carbapenem-susceptible (*n* = 6)		0%	1 - 2	2	2

BMD: broth microdilution

The European Committee on Antimicrobial Susceptibility Testing (EUCAST). Breakpoint tables for interpretation of MICs and zone diameters, Version 12.0, 2022.

Table S2. Comparison of testing methods for the determination of susceptibility of challenge as well as ESBL-negative and carbapenem-susceptible isolates to ceftolozane/tazobactam according to the current EUCAST breakpoints.

Organism (No of isolates)	Assay	Resistance rate	MIC (mg/L)		
			Range	MIC_{50}	MIC_{90}
***Enterobacterales* (*n* = 83)**		48%	≤0.25 - >32	1	>32
K. pneumoniae (*n* = 57)		69%	≤0.25 - >32	>32	>32
Challenge (*n* = 44)		84%	≤0.25 - >32	>32	>32
ESBL-negative, carbapenem-susceptible (*n* = 13)		0%	≤0.25 - 0.5	≤0.25	0.5
E. coli (*n* = 19)		5%	≤0.25 - >32	≤0.25	0.5
Challenge (*n* = 11)	**BMD**	9%	≤0.25 - >32	≤0.25	1
ESBL-negative, carbapenem-susceptible (n = 8)		0%	≤0.25 - ≤0.25	≤0.25	≤0.25
E. cloacae (*n* = 7)		29%	≤0.25 - >32	≤0.25	>32
Challenge (*n* = 3)		67%	0.5 - >32	>32	>32
ESBL-negative, carbapenem-susceptible (*n* = 4)		0%	≤0.25 - ≤0.25	≤0.25	≤0.25
***Enterobacterales* (*n* = 83)**		48%	≤0.25 - >16	0.5	>16
K. pneumoniae (*n* = 57)		67%	≤0.25 - >16	>16	>16
Challenge (*n* = 44)		82%	≤0.25 - >16	>16	>16
ESBL-negative, carbapenem-susceptible (*n* = 13)		0%	≤0.25 - 0.5	≤0.25	0.5
E. coli (*n* = 19)		5%	≤0.25 - >16	≤0.25	0.5
Challenge (*n* = 11)	**Vitek 2**	9%	≤0.25 - >16	≤0.25	1
ESBL-negative, carbapenem-susceptible (n = 8)		0%	≤0.25 - ≤0.25	≤0.25	≤0.25
E. cloacae (*n* = 7)		29%	≤0.25 - >16	≤0.25	16
Challenge (*n* = 3)		67%	1 - >16	16	>16
ESBL-negative, carbapenem-susceptible (*n* = 4)		0%	≤0.25 - ≤0.25	≤0.25	≤0.25
***Enterobacterales* (*n* = 83)**		47%	0.06 - >256	2	>256
K. pneumoniae (*n* = 57)		67%	0.125 - >256	64	>256
Challenge (*n* = 44)		82%	0.25 - >256	>256	>256
ESBL-negative, carbapenem-susceptible (*n* = 13)		0%	0.125 - 1	0.125	1
E. coli (*n* = 19)		5%	0.06 - >256	0.125	1
Challenge (*n* = 11)	**Etest**	9%	0.125 - >256	0.25	2
ESBL-negative, carbapenem-susceptible (n = 8)		0%	0.06 - 0.125	0.125	0.125
E. cloacae (*n* = 7)		29%	0.25 - >256	0.5	>256
Challenge (*n* = 3)		67%	0.5 - >256	>256	>256
ESBL-negative, carbapenem-susceptible (*n* = 4)		0%	0.25 - 0.5	0.25	0.5
***P. aeruginosa* (*n* = 17)**		29%	0.5 - >32	0.5	>32
Challenge (*n* = 11)	**BMD**	45%	0.5 - >32	1	>32
ESBL-negative, carbapenem-susceptible (*n* = 6)		0%	0.5 - 0.5	0.5	0.5
***P. aeruginosa* (*n* = 17)**		29%	0.5 - >16	0.5	>16
Challenge (*n* = 11)	**Vitek 2**	45%	0.5 - >16	1	>16
ESBL-negative, carbapenem-susceptible (*n* = 6)		0%	0.5 - 0.5	0.5	0.5
***P. aeruginosa* (*n* = 17)**		29%	0.5 - >256	1	>256
Challenge (*n* = 11)	**Etest**	45%	0.5 - >256	1	>256
ESBL-negative, carbapenem-susceptible (*n* = 6)		0%	0.5 - 0.5	0.5	0.5

BMD: broth microdilution

The European Committee on Antimicrobial Susceptibility Testing (EUCAST). Breakpoint tables for interpretation of MICs and zone diameters, Version 12.0, 2022.

Table S3. Method comparison agreement, error categories and acceptance criteria as per the ISO standard 20776-2.

Agreement or error category	Acceptance criteria
Category Agreement (CA)<br>Percentage of isolates (%) producing the same interpretive category result (susceptible or resistant) as compared to the reference broth microdilution (BMD) method	≥90% CA
Essential Agreement (EA)<br>Percentage of isolates (%) producing MICs that are within ±one serial 2-fold dilution of the BMD method	≥90% EA
Major Error (ME)<br>ME occurred when VITEK 2 AST-GN or Etest categorized a given isolate as resistant and BMD as susceptible (false resistance)	≤3% ME
Very Major Error (VME)<br>VME occurred when VITEK 2 AST-GN or Etest categorized an isolate as susceptible and BMD as resistant (false susceptibility)	≤3% VME

ISO - ISO 20776-2:2021 - Clinical laboratory testing and *in vitro* diagnostic test systems - Susceptibility testing of infectious agents and evaluation of performance of antimicrobial susceptibility test devices - Part 2: Evaluation of performance of antimicrobial susceptibility test devices against reference broth micro-dilution.
